# Autoimmune Gastrointestinal Paralysis: Failure of Conventional Treatment without Immunomodulation

**DOI:** 10.1155/2014/180654

**Published:** 2014-09-18

**Authors:** Craig Weinkauf, Sean McPhillips, Robert Krouse, Ira Levine

**Affiliations:** ^1^University of Arizona, 1501 N Campbell, Tucson, AZ 85721, USA; ^2^Southern Arizona VA Health Care System, 3601 S 6th, Tucson, AZ 85723, USA

## Abstract

The treatment of the rare enteric nervous system (ENS) manifestations of paraneoplastic syndromes, which are most frequently associated with small cell lung cancer (SCLC), is poorly understood and described. Patients with neuroendocrine-derived tumors can develop B-cell reactivity towards the tumor with cross-reactivity for neurons located in the submucosal and myenteric ganglia of the ENS. The ensuing autoimmune neuritis causes aperistalsis and severe gastrointestinal (GI) dysfunction. Immune-directed therapy is not the standard of care but may be paramount for patient recovery. Our patient, a 63-year-old man with recent symptoms of esophageal dysmotility and newly diagnosed SCLC was hospitalized with nausea, emesis, and constipation. After an extensive work-up that included laparoscopy and celiotomy with bowel resection, we diagnosed what we refer to as Autoimmune Paraneoplastic Chronic Intestinal Pseudoobstruction (AP-CIPO). Unlike the few clinically similar reports, SCLC and AP-CIPO were diagnosed in our patient within weeks of each other, which presented the dilemma of treating the two processes simultaneously. In this report, we review the relevant literature and describe our patient's course. We believe standard chemotherapy is not effective treatment for AP-CIPO. Based on evidence discussed herein, we suggest initiating autoimmune-directed therapy before or simultaneous with cancer-directed therapy.

## 1. Background

Impaired intestinal motility, chronic intestinal pseudoobstruction (CIPO), paraneoplastic intestinal pseudoobstruction, and similar names are found in the few case reports that refer to a rare clinical condition associated with certain cancers that result in severe GI dysmotility. This disease is most commonly associated with small cell lung cancer (SCLC) [1]. Evidence indicates that the disease is caused by B lymphocytes that recognize tumor antigens and cross-react with cells of the normal ENS, resulting in an autoimmune inflammatory response causing neuritis and ENS cell death. This precludes enteral feeding because the gut is nonfunctional and mandates total parenteral nutrition (TPN) in a patient population already under significant metabolic stress and potentially undergoing chemotherapy treatment for the cancer. Appropriate patient care and treatment are often further complicated by the delayed diagnosis because it is a rare disease with no defined treatment. In agreement with the varied nomenclature in other case reports for these pseudoobstructive disorders, but to better reflect our growing understanding of disease etiology, we refer to this GI disease as Autoimmune Paraneoplastic Chronic Intestinal Pseudoobstruction (AP-CIPO).

## 2. Case Presentation

A 63-year-old male with COPD and a long smoking history presented with new symptoms of left sided facial pain and increasing shortness of breath. Biopsy of a left supraclavicular lymph node showed metastatic SCLC (immunohistochemistry positive for thyroid transcription factor-1 (TTF-1), synaptophysin, and chromogranin). His PET-CT showed a large fludeoxyglucose-avid lesion in the left upper lobe and FDG-avid left supraclavicular lymph nodes; outpatient chemotherapy was arranged. He was readmitted to the hospital two weeks later with a two-day history of diffuse abdominal pain, nausea, and bilious emesis. A computed tomography (CT) scan showed distended loops of small bowel without a clear transition point. No hernias were noted and he had no prior abdominal surgery. He was treated conservatively for partial small bowel obstruction versus ileus with a nasogastric tube (NGT). After continued high NGT output and lack of bowel movement for a week, he was taken to the operating room for a diagnostic laparoscopy. The large intestine appeared grossly normal; 20–30 cm of ileum proximal to the ileocecal (IC) valve was peristalsing but remained full of digestive material. Proximal to this, there was mildly dilated small bowel that was aperistaltic. At this time, our differential was ileus versus obstruction at the IC valve. The procedure was terminated without further intervention because there was no clear indication for bowel resection or bypass. Erythromycin was added to his regimen of senna and bisacodyl suppository for threedays without effect. A GI series showed no progression of contrast through the intestines for one week after the initial operation. The patient was then taken back to the operating room for an ileocecectomy with resection of 5–10 cm of bowel proximally and distally with primary anastamosis. All of the inspissated fecal material was either included in the specimen or milked out of the remaining bowel. Initial tissue microscopy showed no pathology.

**Figure 1:**
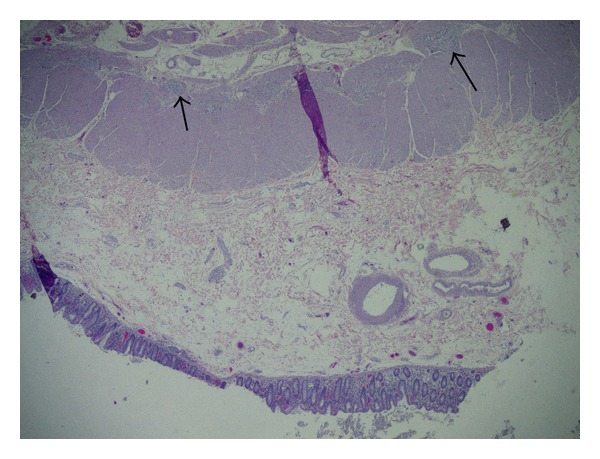
Low-power cross section showing lymphocytic infiltrate into the myenteric plexus.

**Figure 2:**
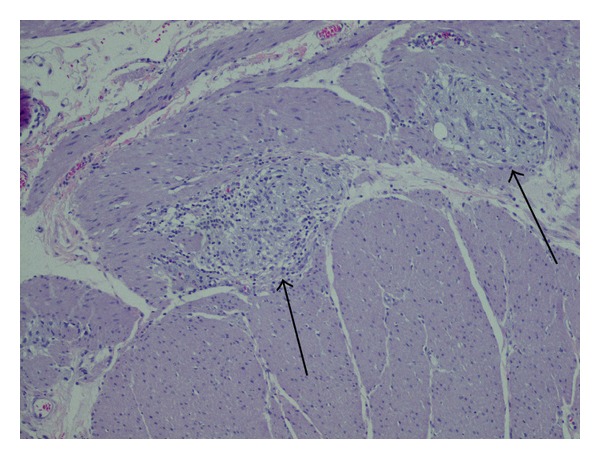
Mid-power cross section showing lymphocytic infiltrate into the myenteric plexus.

Postoperatively, the patient was started on TPN. He remained stable but had no signs of bowel function recovery despite treatment with methylnaltrexone, erythromycin, metoclopramide, senna, enemas, and bisacodyl suppositories. The patient developed paroxysmal atrial flutter and atrial fibrillation and his blood pressure and heart rate were markedly labile. Initially, intravenous metoprolol and later oral scheduled metoprolol controlled his heart rate. After his atypical course, we requested reexamination of the proximal portion of resected bowel (the region that was aperistaltic). This showed lymphoplasmacytic infiltrate consistent with myenteric ganglioneuritis (Figures 1 and 2). This supported our new working diagnosis of AP-CIPO. Based on the limited literature available to guide therapy, a trial of octreotide (100 mcg BID) was initiated and was increased to 200 mcg TID [2]. There was no clinical benefit of octreotide during his month course. Later, a lab showed anti-Hu antibodies, further reinforcing the diagnosis of AP-CIPO. After discussion with the patient and his oncologists, it was decided that chemotherapy for the lung cancer would be initiated. His regimen was started with a dexamethasone taper, cisplatin, and etoposide.

**Figure 3:**
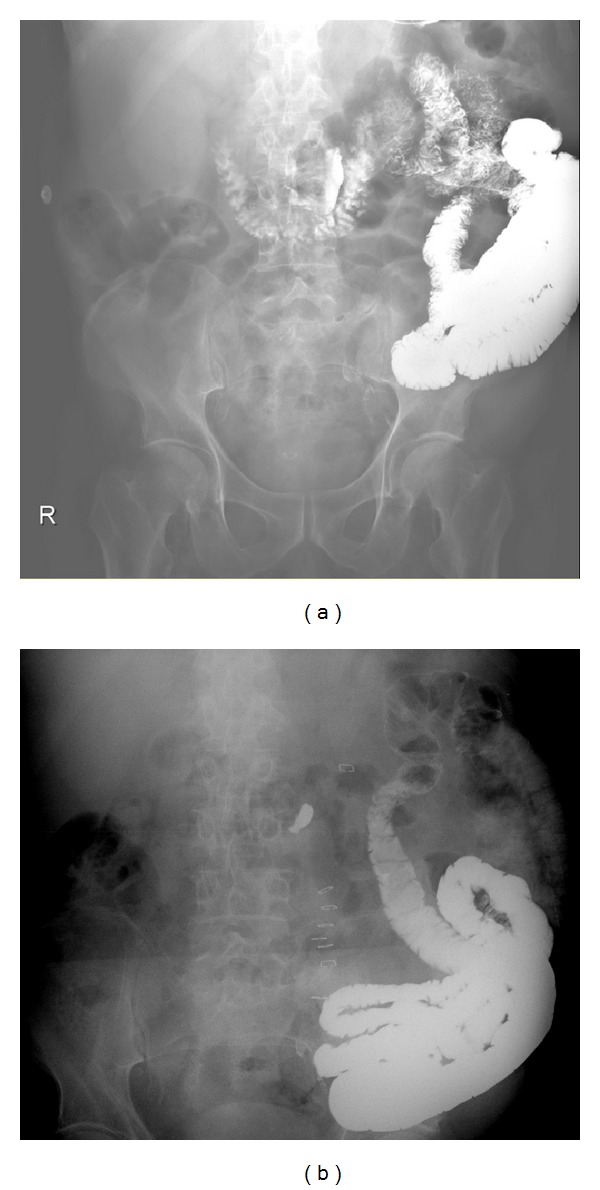
(a) Abdominal X-ray taken three days before the patient's small bowel resection. (b) Abdominal X-ray taken nine days after the patient's small bowel resection. Note how the contrast has not progressed and remains in the distal jejunum and ileum.

Our patient never regained bowel function with chemotherapy over the following month. Abdominal X-ray showed little to no progression of contrast through his intestines (Figure 3). He acutely decompensated 7 weeks after his admission with new onset bacteremia and pneumonia. Eight weeks from his admission, care was withdrawn and the patient died.

## 3. Discussion 

### 3.1. Disease Background and Diagnosis

Paraneoplastic syndromes can affect any organ system in the body. Neurological manifestations are the least common and are almost always associated with SCLC [3]. GI manifestations of paraneoplastic syndromes can include dysmotility of the esophagus, stomach, and small and large bowel [4]. In these patients, it is thought that B lymphocytes educated to recognize tumor antigens become cross-reactive with normal nervous tissue. Several antibodies are thought to cause AP-CIPO including ANNA-1 (type 1 antineuronal nuclear antibody), type 1 Purkinje cell cytoplasmic antibody (PCA-1 or anti-Yo), or N-type calcium channel antibodies [5]. Our patient was positive for anti-Hu antibodies (also called ANNA-1). B-cells produce anti-Hu antibodies that react with a family of RNA-binding neural proteins (Hel and Hu proteins) [6]. Autoimmune destruction of the ENS ensues and with a lymphoplasmacytic infiltrate in the myenteric and submucosal plexi with a paucity of ganglion cells [6]. An alternative to this autoimmune hypothesis is a SCLC paraneoplastic syndrome in which the cancer produces hormones or peptides that inhibit GI function [2].

The diagnosis of AP-CIPO is typically delayed and patients often undergo extensive evaluations and multiple noncurative bowel resections [7–9] as the GI dysmotility is refractory to conventional treatments. In addition, patients often have other manifestations of autonomic nervous system dysregulation including hypothermia, hypoventilation or, as we saw in our patient, cardiac arrhythmias, and blood pressure instability. The diagnosis of AP-CIPO is made based on the clinical symptoms noted above in conjunction with pathology results showing destruction of enteric nervous tissue and the presence of serum anti-Hu antibodies. Scintigraphic gastric emptying, esophageal manometry [5], thermoregulatory sweat test (testing autonomic nervous function), and postural hypotension evaluation can be helpful clinical findings to support a diagnosis of AP-CIPO [10].

### 3.2. Treatment

The treatment of AP-CIPO is poorly defined. We found four case reports in the literature describing different treatment regimens. One manuscript reported success with octreotide treatment [2]. Another reported marginal results with a regimen including prednisone, azathioprine, and budesonide [11]. The other two reported success with B-cell-directed therapy using rituximab [12] or rituximab and cyclophosphamide [9]. Rituximab is an anti-CD20 antibody that selectively depletes B cells (not plasma cells); cyclophosphamide has broader inhibition of dividing T cells and B cells. One case found resolution of gastroparesis after resection of a primary retroperitoneal leiomyosarcoma believed to be caused by a similar autoimmune mechanism [13]. Our patient differed from these other four reports because his cancer was newly diagnosed and the indication for initiating chemotherapy was clear. Some of the previous cases had already been treated with chemotherapy months to years previously and, although recurrence was eventually suspected, their presenting problem was only GI dysfunction [2, 9].

In our patient, the decision was made to start chemotherapy without further delay and without directed immunosuppressive therapy. Our patient had no GI improvement with octreotide or through effects of cisplatin and etoposide directly on the immune system or indirectly through tumor suppression. It is conceivable that the added stress of being malnourished without GI function contributed to our patient's death and initiating immune-targeted therapy could have improved our patient's outcome.

Bowel resection is another key issue in patients with AP-CIPO. All of the reported cases have described bowel resection during their diagnosis/treatment course. As with our patient, bowel resection was a response to failed conservative management of presumed partial small bowel obstruction. Bowel resection likely has no initial role in the initial treatment of AP-CIPO. However, after autoimmune-directed medical therapy, it is possible that some sections of bowel may not recover in every patient, and, guided by nerve function testing or direct visualization, bowel resection may be beneficial.

## 4. Conclusion

Autoimmune Paraneoplastic Chronic Intestinal Pseudoobstruction (AP-CIPO) is a very rare disease seen in patients with nerve cell-derived tumors, most commonly SCLC. Although recognizing the disease is difficult due to its rarity, diagnosis can be made based on clinical findings (symptoms of bowel obstruction and autonomic nervous system dysfunction), intestinal pathology, and autoantibody testing. Based on disease mechanisms, our experience, and the combined experience gained from the few case reports available, we would promote treating AP-CIPO with autoimmune-directed therapy using rituximab.

## Abbreviations

AP-CIPO: Autoimmune paraneoplastic chronic intestinal pseudoobstruction
PET-CT: Positron emission tomography-computerized tomography
TPN: Total parenteral nutrition
GI: Gastrointestinal
ENS: Enteric nervous system
IC: Ileocecal
SCLC: Small cell lung cancer
NGT: Nasogastric tube
CT: Computed tomography
ANNA-1: Type 1 antineuronal nuclear antibody
PCA-1: Type 1 Purkinje cell cytoplasmic antibody.
